# Effect of Kaempferol and Its Glycoside Derivatives on Antioxidant Status of HL-60 Cells Treated with Etoposide

**DOI:** 10.3390/molecules27020333

**Published:** 2022-01-06

**Authors:** Magdalena Kluska, Michał Juszczak, Jerzy Żuchowski, Anna Stochmal, Katarzyna Woźniak

**Affiliations:** 1 Department of Molecular Genetics, Faculty of Biology and Environmental Protection, University of Lodz, 90-236 Lodz, Poland; magdalena.kluska@edu.uni.lodz.pl (M.K.); michal.juszczak@edu.uni.lodz.pl (M.J.); 2 Department of Biochemistry and Crop Quality, Institute of Soil Science and Plant Cultivation, State Research Institute, 24-100 Pulawy, Poland; jzuchowski@iung.pulawy.pl (J.Ż.); asf@iung.pulawy.pl (A.S.)

**Keywords:** kaempferol, kaempferol derivatives, etoposide, oxidative stress, gene expression, SOD activity, glutathione

## Abstract

Kaempferol is a well-known antioxidant found in many plants and plant-based foods. In plants, kaempferol is present mainly in the form of glycoside derivatives. In this work, we focused on determining the effect of kaempferol and its glycoside derivatives on the expression level of genes related to the reduction of oxidative stress—*NFE2L2*, *NQO1*, *SOD1*, *SOD2*, and *HO-1*; the enzymatic activity of superoxide dismutases; and the level of glutathione. We used HL-60 acute promyelocytic leukemia cells, which were incubated with the anticancer drug etoposide and kaempferol or one of its three glycoside derivatives isolated from the aerial parts of *Lens culinaris* Medik.—kaempferol 3-*O*-[(6-*O-E*-caffeoyl)-β-d-glucopyranosyl-(1→2)]-β-d-galactopyranoside-7-*O*-β-d-glucuropyranoside (P2), kaempferol 3-*O*-[(6-*O-E-p*-coumaroyl)-β-d-glucopyranosyl-(1→2)]-β-d-galactopyranoside-7-*O*-β-d-glucuropyranoside (P5), and kaempferol 3-*O*-[(6-*O-E*-feruloyl)-β-d-glucopyranosyl-(1→2)]-β-d-galactopyranoside-7-*O*-β-d-glucuropyranoside (P7). We showed that none of the tested compounds affected *NFE2L2* gene expression. Co-incubation with etoposide (1 µM) and kaempferol (10 and 50 µg/mL) leads to an increase in the expression of the *HO-1* (9.49 and 9.33-fold at 10 µg/mL and 50 µg/mL, respectively), *SOD1* (1.68-fold at 10 µg/mL), *SOD2* (1.72-fold at 10–50 µg/mL), and *NQO1* (1.84-fold at 50 µg/mL) genes in comparison to cells treated only with etoposide. The effect of kaempferol derivatives on gene expression differs depending on the derivative. All tested polyphenols increased the SOD activity in cells co-incubated with etoposide. We observed that the co-incubation of HL-60 cells with etoposide and kaempferol or derivative P7 increases the level of total glutathione in these cells. Taken together, our observations suggest that the antioxidant activity of kaempferol is related to the activation of antioxidant genes and proteins. Moreover, we observed that glycoside derivatives can have a different effect on the antioxidant cellular systems than kaempferol.

## 1. Introduction

Reactive oxygen species (ROS) play an important role in the pathogenesis of many diseases, including cancer, neurodegenerative, the respiratory system, and gastrointestinal tract diseases [1]. A high level of ROS in normal cells contributes to the damage of DNA, lipids, and proteins and then to mutagenesis and transformation [2]. On the other hand, cancer cells are characterized by elevated levels of ROS caused by altered metabolism and increased energy demand [1]. ROS play a role in different stages of cancer development, such as transformation, invasion, and metastasis [3]. Glutathione (γ-glutamyl-cysteinyl-glycine) is a primary low-molecular-weight thiol that plays a key role in antioxidant defense; nutrient metabolism; and the regulation of cellular processes, such as DNA and protein synthesis, gene expression, or apoptosis [4]. Under physiological conditions within the cell, glutathione exists mainly (>99%) in the thiol-reduced form (GSH), but some is present as GSSG (glutathione disulfide), as well as a variety of thioether, mercaptide, or other thioester forms (glutathione S-conjugates). Increased levels of GSH have been associated with chemotherapeutic resistance, e.g., for platinum-containing compounds, alkylating agents (such as melphalan), anthracyclines, doxorubicin, or arsenic, and to ionizing radiation resistance [5]. It was proposed that a decrease in the glutathione level was associated with the sensitizing of leukemia cells to chemotherapy in combination with polyphenols [6]. Treatments with quercetin, apigenin, emodin, rhein, and *cis*-stilbene decrease the GSH level in lymphoid leukemia cell lines (Jurkat and CCRF-CEM) and leads to enhancement of the anticancer activity of topoisomerase II poisons etoposide or doxorubicin. Moreover, in myeloid cell lines (THP-1 and KG1a), quercetin and apigenin reduce the level of glutathione and enhance the apoptotic effect of the drugs [7].

Nuclear factor erythroid 2-like 2 (NFE2L2 or NRF-2) is a key cytoprotective molecule responsible for regulating the expression of antioxidant proteins, including heme oxygenase-1 (HO-1), NAD(P)H:quinone oxidoreductase 1 (NQO1), the catalytic subunit of glutamate-cysteine ligase (GCLC), and multi-drug resistance genes family [8,9,10,11]. It is sequestered in the cytoplasm by Kelch-like ECH-associated protein 1 (Keap1). During oxidative stress, NFE2L2 is released from Keap1 and then translocated into the nucleus. There, along with other transcription factors, NFE2L2 activates the transcription of genes containing an antioxidant response element (ARE) [12]. Unfortunately, NFE2L2 activation triggers antioxidant pathways that protect cancer cells against the effects of chemotherapeutic agents [13]. Moreover, the *NFE2L2* gene is overexpressed in many types of human tumors, such as breast cancer [14] or gastric cancer [15], which contributes to chemoresistance. On the other hand, NFE2L2 silencing increases the sensitivity of cancer cells to chemotherapeutic drugs [16]. NFE2L2 is constitutively active in human acute myeloid leukemia (AML) cells, and these cells possess greater constitutive nuclear levels of NFE2L2 than normal control CD34+ cells [17].

HO-1 catalyzes heme degradation to equimolar amounts of carbon monoxide (CO), iron ions, and biliverdin, which is rapidly converted to bilirubin through the action of biliverdin reductase (BVR). Although the main mechanism of HO-1 activity is related to heme metabolism, HO-1 also plays a role in the cellular response to oxidative stress. HO-1 is upregulated by many different factors, not only by heme but also by nitric oxide, heavy metals, growth factors, cytokines, or modified lipids. Moreover, the HO-1 protein is involved in several other cellular processes, such as cell proliferation, angiogenesis and metastasis, apoptosis, and inflammation [9]. The NAD(P)H quinone oxidoreductase 1 gene (*NQO1*) is a downstream gene of NFE2L2-ARE signaling. The NQO1 enzyme is one of the most important enzymes responsible for antioxidant defense. Its primary function is to catalyze the two-electron reduction of endogenous and exogenous quinones to hydroquinones, removing the electrophilic quinones and bypassing semiquinone radical and ROS generation via redox cycling reactions. Moreover, NQO1 can reduce superoxide in tissues with low levels of superoxide dismutases (SOD) [10]. It was shown that the expression level of the *NQO1* gene may be associated with the proliferation of many tumor cells, including brain cancer and leukemia [18,19,20]. NQO1 controls cell proliferation and apoptosis by stabilizing p53 [21].

Superoxide dismutases (SOD) are one of the most important antioxidative enzymes, which convert superoxide radicals into hydrogen peroxide and molecular oxygen. This enzyme occurs in three isoforms: SOD1 (Cu/Zn SOD) is located in the intermembrane space of the mitochondria, the nucleus, and the cytosol; SOD2 (Mn-SOD) is present in the mitochondrial matrix; and SOD3 (Ec SOD) is a secreted form expressed in the lungs, kidneys, and adipose tissues to prevent oxidative tissue damage. SOD1 is the major intracellular form of SOD, accounting for ~80% of the total SOD protein, and it acts as a scavenger of O_2_^−^ generated by NADPH oxidase (NOX), xanthine oxidase, and cytochrome P450. Moreover, SOD1 scavenges O_2_^−^ in the mitochondrial intermembrane space during electron transport [22]. SOD1 is overexpressed in many types of cancer, including non-small cell lung cancer (NSCLC) [23], breast cancer [24], or nasopharyngeal carcinoma [25]. In addition to O_2_^−^ scavenging, SOD1 and SOD2 are actively involved in modulating diverse cellular processes. H_2_O_2_, the dismutating product of O_2_^−^ by SOD, can act as a second messenger to regulate cellular growth and metabolic processes [22]. It is suggested that SOD can be a novel promising target in cancer therapy [26].

**Figure 1:**
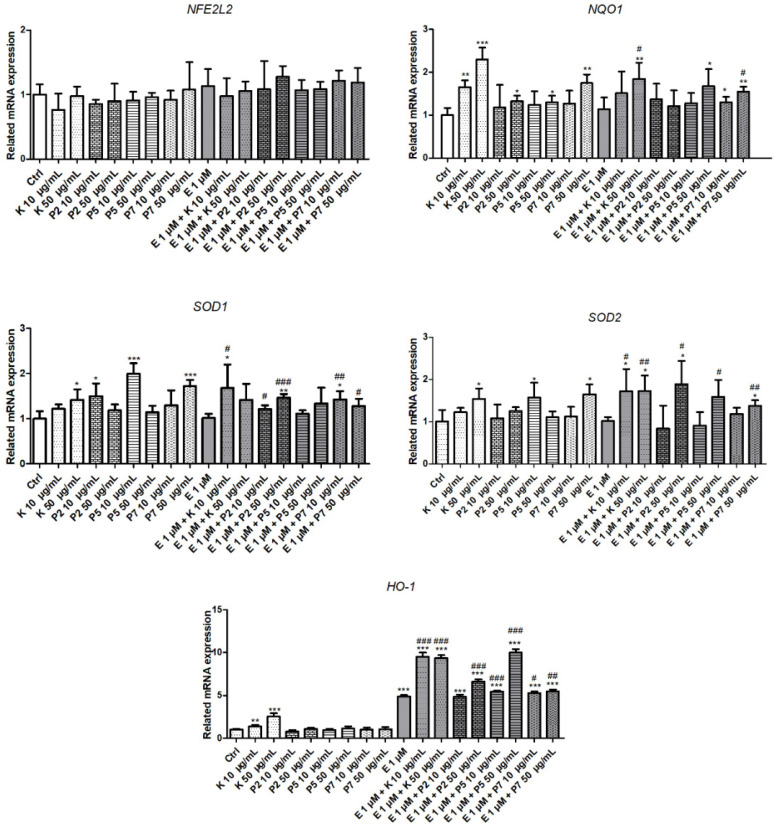
Relative expression of the *NFE2L2*, *NQO1*, *SOD1*, *SOD2*, and *HO-1* genes in HL-60 cells incubated for 24 h at 37 °C with 10–50-µg/mL kaempferol (K), P2, P5, P7, and/or 1-µM etoposide (E). The figure shows the mean results ± SD, *n* = 4; * *p* < 0.05, ** *p* < 0.01, and *** *p* < 0.001 vs. control (Ctrl); # *p* < 0.05, ## *p* < 0.01, and ### *p* < 0.001 vs. etoposide (E).

Kaempferol [3,5,7-trihydroxy-2-(4-hydroxyphenyl)-4H-1-benzopyran-4-one] is a natural flavonoid widely present in tea, grapes, berries, and cruciferous vegetables. It has antioxidant, anticancer, and anti-inflammatory properties [27]. In our previous papers, we analyzed the impact of kaempferol and its glycoside derivatives isolated from *Lens culinaris* Medik. on HL-60 cells and PBMCs (peripheral blood mononuclear cells) treated with etoposide [28,29]. We demonstrated that kaempferol glycosides reduced the DNA damage induced by etoposide in PBMCs, but they did not have an impact on the DNA damage in HL-60 leukemic cells [28]. Previously, we have also shown that kaempferol increases the cytotoxic effect of etoposide in HL-60 cells [29]. On the other hand, kaempferol reduced the level of free radicals generated by etoposide. However, some of the kaempferol derivatives increased the level of free radicals induced by this drug [29]. Due to the fact that the antioxidant status of cancer cells may be of key importance for the effectiveness of anticancer therapy, in the present work, we decided to investigate the effect of kaempferol and its glycoside derivatives on the expression of the *NFE2L2* gene and its downstream genes: *HO-1*, *NQO1*, *SOD1*, and *SOD2* in HL-60 cells treated with etoposide. Here, we used kaempferol glycoside derivatives isolated from aerial parts of *Lens Culinaris* Medik. the same that we used in our last paper (Figure 1B–D [29]). Moreover, here, we determined the activity of SOD and the level of total glutathione content in HL-60 cells treated with etoposide and tested polyphenols.

## 2. Materials and Methods

### 2.1. Reagents

3,4′,5,7-Tetrahydroxyflavone (kaempferol) (K0133), 4′-demethylepipodophyllotoxin 9-(4,6-*O*-ethylidene-β-d-glucopyranoside) (etoposide) (E1383), dimethyl sulfoxide (DMSO), and 5-Sulfosalicylic acid dihydrate (247006) were purchased from Sigma-Aldrich (St. Louis, MO, USA). Kaempferol was dissolved in DMSO and stored at −20 °C. Etoposide was dissolved in methanol. The SOD Assay Kit WST (Lot. SJ972) was purchased from Dojindo Molecular Technologies, Inc. (Kumamoto, Japan). The Glutathione Colorimetric Detection Kit (catalog number: EIAGSHC) was purchased from Thermo Fisher Scientific (Waltham, MA, USA).

### 2.2. Kaempferol Glycosides from the Aerial Parts of Lentil

Kaempferol glycosides: kaempferol 3-*O*-[(6-*O-E*-caffeoyl)-β-d-glucopyranosyl-(1→2)]-β-d-galactopyranoside-7-*O*-β-d-glucuropyranoside (P2), kaempferol 3-*O*-[(6-*O-E-p*-coumaroyl)-β-d-glucopyranosyl-(1→2)]-β-d-galactopyranoside-7-*O*-β-d-glucuropyranoside (P5), and kaempferol 3-*O*-[(6-*O-E*-feruloyl)-β-d-glucopyranosyl-(1→2)]-β-d-galactopyranoside-7-*O*-β-d-glucuropyranoside (P7) were isolated from the aerial parts of *Lens culinaris* Medik., according to the procedure described by Żuchowski et al. (2014) [30]. All isolated kaempferol glycosides were dissolved in 50% DMSO and stored at −20 °C [29].

### 2.3. Cell Preparation and Treatment

The HL-60 (human promyelocytic leukemia) cell line was obtained from the American Type Culture Collection (ATCC) (Manassas, VA, USA). The cells were cultured in flasks at 37 °C in 5% CO_2_ atmosphere in Iscove’s Modified Dulbecco’s Medium (IMDM), 2-mM L-glutamine, and 25-mM HEPES (Lonza, Basel, Switzerland) with 15% inactivated fetal bovine serum (FBS) and penicillin/streptomycin solution (100 U/mL and 100 μg/mL, respectively).

In all experiments, the HL-60 cells were seeded in the culture medium and then incubated at 37 °C with different concentrations (10–50 µg/mL) of kaempferol: kaempferol 3-*O*-[(6-*O-E*-caffeoyl)-β-d-glucopyranosyl-(1→2)]-β-d-galactopyranoside-7-*O*-β-d-glucuropyranoside (P2), kaempferol 3-*O*-[(6-*O-E-p*-coumaroyl)-β-d-glucopyranosyl-(1→2)]-β-d-galactopyranoside-7-*O*-β-d-glucuropyranoside (P5), and kaempferol 3-*O*-[(6-*O-E*-feruloyl)-β-d-glucopyranosyl-(1→2)]-β-d-galactopyranoside-7-*O*-β-d-glucuropyranoside (P7). The cells were also treated with etoposide (1 µM). The final concentration of DMSO and methanol in the samples did not exceed 0.5% [29].

### 2.4. Gene Expression Analysis

To analyze the gene expression, the cells (0.5 × 10^5^) were seeded in 6-well plates and incubated with 1-µM etoposide and/or 10–50-µg/mL kaempferol, P2, P5, and P7 at 37 °C for 24 h. Afterwards, the cells were collected, and the total RNA from each sample was extracted using the Universal RNA Purification Kit (EurX, Gdansk, Poland), according to the manufacturer’s instructions. Reverse transcription and real-time PCR reactions were performed using the SensiFAST™ Probe No-ROX One-Step Kit (Bioline, London, UK) on the CFX96 C1000 real-time system (Bio-Rad, Hercules, CA, USA). The relative expression was evaluated using the TaqMan gene expression assay for *HO-1*: Hs00157965_m1, *NFE2L2*: Hs00975961_m1, *NQO1*: Hs00168547_m1, *SOD1*: Hs00533490_m1, *SOD2*: Hs00167309_m1, and *GAPDH*: Hs02786624_m1 (Thermo Fisher Scientific, Waltham, MA, USA). The *GAPDH* gene was utilized as a reference gene. Each assay was performed in quadruplicate. The relative gene expression was calculated as the fold change according to the control sample based on the double-delta Ct method.

### 2.5. Superoxide Dismutase (SOD) Activity

To measure the activity of SOD, the cells were seeded in 6-well plates and incubated with 1-µM etoposide and/or 10–50-µg/mL kaempferol, P2, P5, and P7 at 37 °C for 24 h. Then, the cells were collected, and the cell lysates were prepared. The cells (1 × 10^6^) were centrifuged at 250× *g* for 10 min at 4 °C. Next, the supernatant was discarded, and the cell pellet was resuspended in ice-cold PBS. Then, the cells were centrifuged again at 250× *g* for 10 min at 4 °C. The supernatant was discarded, and the cells were resuspended in 0.5 mL of PBS and sonicated under ice for 30 s using the 4710 Series Ultrasonic Homogenizer (Cole-Parmer Instrument Co., Chicago, IL, USA). After that, samples were centrifuged at 1500× *g* for 10 min at 4 °C. The supernatant was collected, transferred into 96-well plates, and the SOD activity was measured. To determine the SOD activity, the SOD Assay Kit WST was used according to the manufacturer’s procedures (Dojindo Molecular Technologies, Inc., Kumamoto, Japan). The plates were incubated for 20 min at 37 °C, and the absorbance at 450 nm was measured using a Synergy HT microplate reader (Biotek, Bad Friedrichshall, Germany). Each assay was performed in triplicate. Then, the SOD activity was calculated using the following Equation (1):

SOD activity (inhibition rate%) = [(A_blank1_ − A_blank3_) − (A_sample_ − A_blank2_)]/(A_blank1_ − A_blank3_) × 100 (1)

A—absorbance

The data were normalized to the negative control, which was assigned as 100% of the SOD activity.

### 2.6. Determination of Total Glutathione Content

The total glutathione (GSH + GSSG) content was determined using the Glutathione Colorimetric Detection Kit (Thermo Fisher Scientific, Waltham, MA, USA). HL-60 cells were incubated with 1-µM etoposide and/or 10–50-µg/mL kaempferol, P2, P5, and P7 in culture flasks for 24 h. After the incubation, cells were collected and centrifuged at 800× *g* for 10 min at 4 °C. Then, the cells were washed by centrifugation in ice-cold PBS at 800× *g* for 10 min at 4 °C. The (4 × 10^6^) cells were resuspended in ice-cold 5% 5-sulfosalicylic acid solution and sonicated under ice for 30 s using the 4710 Series Ultrasonic Homogenizer (Cole-Parmer Instrument Co., Chicago, IL, USA). Next, the homogenate was incubated for 10 min at 4 °C and centrifuged at 17,000× *g* for 10 min at 4 °C. The supernatant was collected, transferred into 96-well plates, and assayed for glutathione, according to the manufacturer’s instructions. After 5 min of incubation at room temperature, the absorbance was measured at 405 nm using a Synergy HT microplate reader (Biotek, Bad Friedrichshall, Germany). Each assay was performed in triplicate. The data were normalized to the negative control, which was assigned as 100% of the GSH level (GSH + GSSG).

### 2.7. Statistical Analysis

Expression of the genes was calculated based on the double-delta Ct method. The data were presented as the mean ± SD from 4 independent experiments. In the case of SOD activity and the glutathione level, the data were presented as the mean ± SD from 3 independent experiments. Statistical differences were determined by one-way ANOVA with the post hoc Tukey’s multiple comparison test and by the Student’s two-tailed *t*-test. Statistics were performed using GraphPad Prism 5 (GraphPad Software Inc., La Jolla, CA, USA). The differences were considered to be statistically significant when the *p*-value was less than 0.05.

## 3. Results

### 3.1. Antioxidant Defense Gene Expression

We determined the expression of antioxidant defense genes: *NFE2L2*, *HO-1*, *NQO1*, *SOD1*, and *SOD2* in HL-60 cells incubated with a combination of etoposide and kaempferol or its glycoside derivatives (Figure 1). We did not observe any impact of etoposide; kaempferol; or its glycoside derivatives (P2, P5, and P7) on the expression level of the *NFE2L2* gene. Among the examined genes, etoposide did not change the expression profiles of *NQO1*, *SOD1*, and *SOD2*. We noticed that etoposide increased the expression of the *HO-1* gene 4.9-fold (*p* < 0.001) at a 1-µM concentration. Kaempferol also induced a 1.4-fold and 2.5-fold increase of *HO-1* gene expression at the 10-µg/mL and 50-µg/mL concentrations (*p* < 0.05 and *p* < 0.001, respectively). Moreover, the co-treatment with kaempferol significantly increased the expression of *HO-1* in comparison to cells incubated only with etoposide, from 4.9-fold to 9.5-fold and 9.3-fold at the 10-µg/mL and 50-µg/mL concentrations, respectively (*p* < 0.001). We did not observe any changes in the *HO-1* gene expression after incubation with all kaempferol glycoside derivatives. Interestingly, we observed an increase in the *HO-1* expression in cells co-treated with etoposide and kaempferol derivatives. The greatest increase was recorded in the case of 50-µg/mL P5, from 4.9-fold to 10-fold compared to etoposide (E) (*p* < 0.001). A less significant increase occurred also in the case of co-incubation with derivatives P2 at 50 µg/mL, P5 at 10 µg/mL, and P7 at both concentrations.

We observed that incubation with kaempferol increased the *NQO1* gene expression in both used concentrations (*p* < 0.01 and *p* < 0.001, respectively) (Figure 1). Moreover, all three tested kaempferol glycosides, at the concentration of 50 µg/mL, also increased the *NQO1* expression. The co-treatment of cells with kaempferol or its glycosides and etoposide basically did not affect the expression of the *NQO1* gene. We observed only a slight increase (*p* < 0.05) of *NQO1* expression in cells co-treated with etoposide and 50-µg/mL kaempferol and 50-µg/mL P7.

The next genes we examined were superoxide dismutases *SOD1* and *SOD2*. We noticed that 50-µg/mL kaempferol slightly increased the levels of both the *SOD1* and *SOD2* genes (1.4-fold and 1.5-fold, respectively) (*p* < 0.01) (Figure 1). In the case of P2, we observed only a slight increase in the *SOD1* expression in lower concentrations (*p* < 0.05). The glycoside derivative of kaempferol P5 increased *SOD1* and *SOD2* only in the 10-µg/mL concentration (*p* < 0.001 and *p* < 0.05, respectively), while P7 increased the expression of both *SOD1* and *SOD2* in a higher concentration (*p* < 0.001 and *p* < 0.05, respectively). We observed the increase of *SOD1* expression in cells co-incubated with etoposide and kaempferol (10 µg/mL), P2 (10–50 µg/mL), and P7 (10–50 µg/mL) glycoside derivatives compared to drug-only treated cells. We also noted that the co-treatment of cells with etoposide and kaempferol significantly increased the expression of the *SOD2* gene (*p* < 0.05 and *p* < 0.01). The increase in *SOD2* expression also occurs in the case of cells co-incubated with etoposide and all three tested kaempferol derivatives at the concentration of 50 µg/mL (1.9-fold, 1.6-fold, and 1.4-fold, respectively).

A summary of the results of the antioxidant defense genes expression after incubation of the HL-60 cells with etoposide and polyphenols is presented in Table S1.

### 3.2. SOD Activity

**Figure 2:**
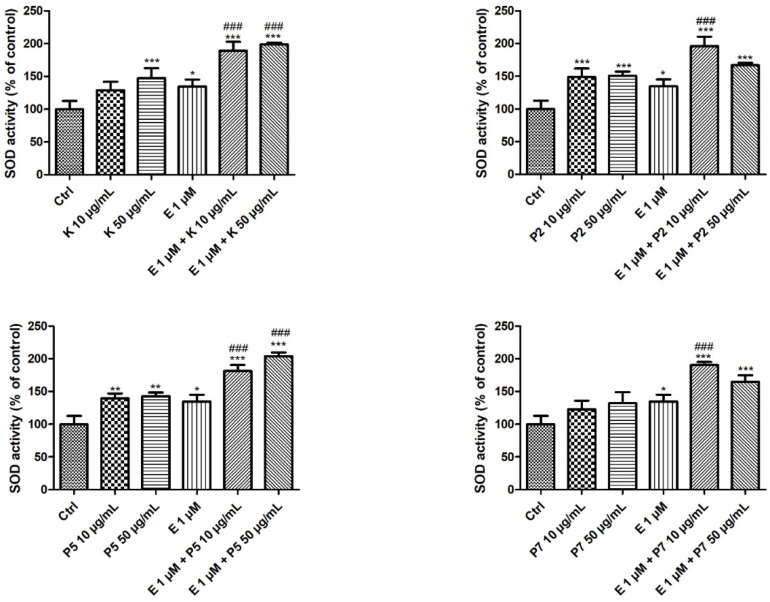
Superoxide dismutase activity in HL-60 cells incubated for 24 h at 37 °C with 10–50-µg/mL kaempferol (K), P2, P5, P7, and/or 1-µM etoposide (E). The figure shows the mean results ± SD, *n* = 3; * *p* < 0.05, ** *p* < 0.01, and *** *p* < 0.001 vs. control (Ctrl); ### *p* < 0.001 vs. etoposide €. Data were normalized to the negative control, which was assigned as 100% of the SOD activity.

The next step was to examine the enzymatic activity of SOD. For this purpose, we performed the determination of the enzymatic activity using the WST-1 solution. Firstly, we noticed that 1-µM etoposide induced a slight increase in the SOD enzyme activity (*p* < 0.05) (Figure 2). As in the case of gene expression, we did not observe any changes in the cells incubated with 10-µg/mL kaempferol in the enzymatic activity test. Moreover, we noticed that 50-µg/mL kaempferol increased the SOD activity to 147% in comparison to the control cells (*p* < 0.001) (Figure 2). Similar activity to kaempferol was shown by its two glycoside derivatives P2 and P5. They increased the enzymatic activity of SOD in HL-60 cells at both concentrations used. On the other hand, the P7 compound did not affect the SOD activity. Interestingly, we observed that all the tested polyphenols increased the SOD activity in cells incubated with etoposide. In the case of kaempferol and derivative P7, the increase in SOD activity in the cells incubated simultaneously with etoposide occurred at both tested concentrations (*p* < 0.001). In contrast, P2 and P7 only increased the activity of SOD in drug-treated cells at a lower concentration (*p* < 0.001).

### 3.3. Total Glutathione Level

**Figure 3:**
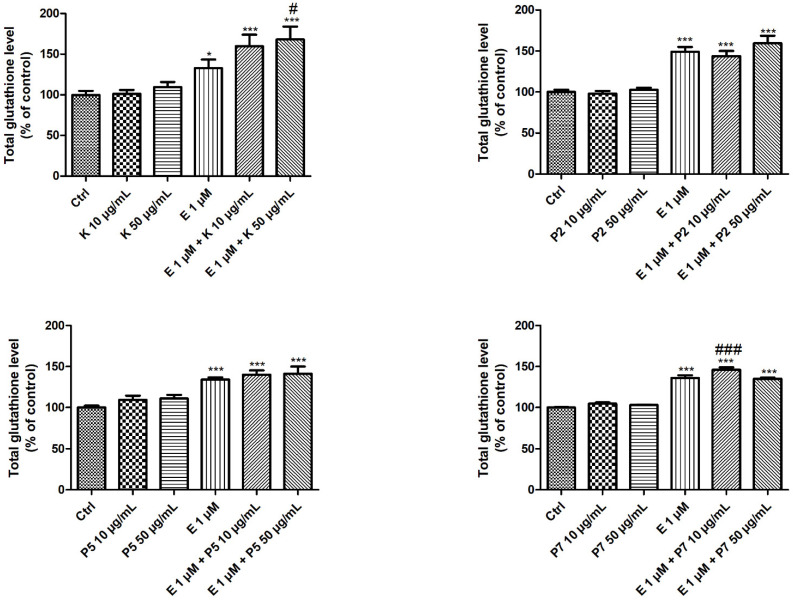
The total glutathione level in HL-60 cells incubated for 24 h at 37 °C with 10–50-µg/mL kaempferol (K), P2, P5, P7, and/or 1-µM etoposide (E). The figure shows the mean results ± SD, *n* = 3; * *p* < 0.05, and *** *p* < 0.001 vs. control (Ctrl); # *p* < 0.05, and ### *p* < 0.001 vs. etoposide (E). Data were normalized to the negative control, which was assigned as 100% of the glutathione level.

We also determined the level of total intracellular glutathione using the colorimetric kit, which was designed to measure the glutathione (GSH) and oxidized glutathione (GSSG) content. Firstly, we noticed that the treatment of HL-60 cells for 24 h with 1-µM etoposide increased the level of total intracellular glutathione (Figure 3). Furthermore, both kaempferol and its glycoside derivatives did not affect the level of glutathione in HL-60 cells. However, we noticed that the co-incubation of cells with 1-µM etoposide and kaempferol at the concentration of 50 µg/mL or derivative P7 at the concentration of 10 µg/mL increased the level of total glutathione in HL-60 cells in comparison to cells treated only with etoposide (*p* < 0.05 and *p* < 0.001, respectively). Additionally, both derivatives P2 and P5 had no effect on the glutathione level in cells incubated with etoposide (Figure 3).

## 4. Discussion

The use of anticancer therapy based on chemotherapeutic agents is associated with the occurrence of side effects caused by the damage of normal cells, such as cardiotoxicity, nephrotoxicity, or leukemia. Polyphenols can protect normal cells from the harmful effects of oxidative stress induced by oxidants, including anticancer drugs. In this paper, we investigated the effect of kaempferol and its glycosides on the etoposide activity in HL-60 cells. Many studies have shown that kaempferol or its derivatives have a beneficial effect on normal cells. For example, a 24-h pre-incubation of human normal lung epithelial (L132) and liver (L02) cells with kaempferol and 3-*O*-methyl quercetin isolated from *S. anacardium* significantly reduced H_2_O_2_-induced stress and increased the expression of NFE2L2, *p*-p38, catalase, and superoxide dismutase-2 [31]. Flavonoids isolated from *Petasites japonicus*, which included, among others, kaempferol-3-*O*-(6″-acetyl)-β-d-glucoside and kaempferol-3-*O*-β-d-glucoside, activated the *NRF-2* and *HO-1* genes [32]. The activation of these pathways protected human dermal fibroblasts (HDF) and human epidermal keratinocyte cells (HEKC) against UVB radiation. Kaempferol protected the auditory cells of mice HEI-OC1 (House Ear Institute-Organ of Corti 1) from apoptosis induced by cisplatin. Its protective effect was based on inducing the expression of the *HO-1* gene, increasing the cellular level of GSH, and the expression of γ-glutamate-cysteine ligase (GCL) [33]. Studies conducted on Balb/C mice have revealed that kaempferol improves cisplatin-induced nephrotoxicity [34]. The ameliorating effect of kaempferol was due to a reduction of oxidative stress, inflammation, and apoptosis via inhibiting the MAPK and NF-κB cascade and upregulation of the NRF-2/HO-1 levels. Moreover, it was shown that kaempferol enhanced the gene expression of thioredoxin reductase 1 (*TXNR1*) and thioredoxin (*TXN*) in normal human keratinocytes. Kaempferol and quercetin also increased the activity of thioredoxin reductase in these cells [35].

Mutations and/or upregulation of the *NFE2L2* gene are observed in many human tumors and are associated with the resistance of cells to chemotherapy [13]. Somatic mutations in the coding region of *NFE2L2* occur especially in patients with a history of smoking or suffering from squamous cell carcinoma and contribute to poor treatment prognosis [36]. NFE2L2 overexpression is associated with a higher frequency of gene mutation and instability-dependent drug resistance in AML patients. In vitro studies have shown that NFE2L2 overexpression protected the AML cells from apoptosis induced by cytarabine. Moreover, this overexpression inhibited MutS Homolog 2 (MSH2) protein expression, which caused a DNA mismatch repair deficiency and induced gene instability-dependent drug resistance in AML [37]. The knockdown of the *NFE2L2* gene in AML cell lines THP1 and U937 sensitized them to the daunorubicin and arsenic trioxide treatment by reducing the reactive oxygen scavenging capacity and downregulation of the target antioxidant genes. Additionally, treatment with brusatol is a pharmacological inhibitor of NFE2L2 and improved the sensitivity of AML cell lines to daunorubicin, arsenic trioxide, and cytarabine [38]. The combination of compounds that inhibit the expression of NFE2L2 with classic anticancer drugs may be a promising approach in the treatment of AML. AML is the most common type of leukemia among adults. The development of the disease is associated with the occurrence of chromosomal abnormalities that lead to the abnormal differentiation of hematopoietic stem cells and accumulation of abnormal blasts in the bone marrow [39]. AML is characterized by a refractory nature and poor prognosis; therefore, the treatment of this disease remains demanding. On the other hand, NFE2L2 controls the basal and induced expression of an array of antioxidant response element-dependent genes. By this way, it is involved in the protection of the body against cancer, but at the same time, it is overexpressed in different tumors, thus resulting in a pro-survival phenotype that favors tumor growth and resistance to oxidants and oncotherapy [40].

Many substances of plant origin, such as polyphenols, can modulate the expression of the *NFE2L2* gene and could be used with chemotherapy to increase the therapeutic effect by sensitizing cells to anticancer drugs. The combination of quercetin and vitamin C decreased the expression of NFE2L2 in both the mRNA and protein levels in the MDA-MB 231, MCF-7, A549, and MDA-MB 468 breast cancer cell lines [41]. In our study, we did not observe any changes in *NFE2L2* gene expression in HL-60 cells treated with etoposide, kaempferol, and its glycosides or with the combination of these compounds (Figure 1). However, we noticed the upregulation of the *NFE2L2* downstream genes *NQO1*, *HO-1*, *SOD1*, and *SOD2* (Figure 1). In NSCLC lung cancer cells, treatment with 25-µM kaempferol inhibited the NFE2L2 signaling pathway by reduction of both the NFE2L2 mRNA and protein levels [42]. Moreover, incubation with kaempferol did not change the level of NF-κB p65 and phospho NF-κB p65 in NSCLC cells. This study also revealed that kaempferol after 24 h of treatment reduced the expression of the *NQO1*, *HO-1*, *GST*, and *AKR1C1* genes, which were dependent on *NFE2L2*. On the other hand, an extract from graviola leaves—rich in kaempferol-rutinoside—increased the expression of the *NFE2L2* gene in HepG2 liver cancer cells but did not affect the expression of the *HO-1* gene [43].

Many studies have indicated the important role of SOD in the pathogenesis and progression of cancer and the possibility of using the inhibition of its expression in therapy. The elevated activity of SOD1 is associated with NSCLC cell proliferation, migration, and invasion. The inhibition of SOD1 expression leads to the promotion of apoptosis and cell cycle arrest in these cells [23]. The knockdown of *SOD1* in nasopharyngeal carcinoma cells reduced its growth and induced apoptosis [25]. SOD1 regulates cell death and differentiation in myeloid leukemia cell lines K562, MEG-01, TF-1, and HEL [44]. Additionally, the inhibition or silencing of *SOD1* increased the cell death induced by PMA (phorbol 12-myristate 13-acetate). We observed that kaempferol and its derivatives increased the expression of the *SOD1* and *SOD2* genes (Figure 1) and elevated the activity of SOD in HL-60 cells (Figure 2). Zhao and co-workers showed that kaempferol at the concentration of 10 µg/mL increased the SOD2 activity in the human colorectal cancer cell line (Caco-2) [45]. Kaempferol at concentrations from 5 to 25 µM increased the SOD activity in 3T3-L1 mouse fibroblast cells [46]. In this paper, we showed that kaempferol and its three tested glycoside derivatives increased the expression of the *SOD1* and *SOD2* genes and the activity of SOD in HL-60 cells co-incubated with etoposide (Figure 1 and Figure 2). On the other hand, it also seems possible that kaempferol can stimulate enzyme activity through epigenetic mechanisms, including miRNAs [47]. This is probably why we showed an increase in SOD activity in the case of P5 at a concentration of 50 µg/mL (Figure 2), in which we did not observe expression of the *SOD1* and *SOD2* genes (Figure 1).

Additionally, other polyphenols can act synergistically with chemotherapeutics by modulating SOD activity. Apigenin inhibited SOD activity in human cervical epithelial carcinoma cells (HeLa) but did not alter the SOD protein level [48]. This inhibition of SOD activity contributed to the increase in the ROS level in the cells and sensitized them to paclitaxel-induced apoptosis. A combination of fluorouracil (5-FU) and natural polymethoxyflavonecastin led to an increase in apoptosis in the mouse leukemia cell line (WEHI-3) [49]. Moreover, this effect was associated with an increase in caspase-3, caspase-8, and caspase-9 activity. The combined treatment also enhanced the expression of Cu/Zn SOD and inhibited the activity of catalase.

Studies conducted on the HL-60 cell line cytarabine (Ara-C)-resistant sub-line HL-60R and on samples collected from patients suffering from AML indicate that an increased level of *HO-1* gene expression is associated with resistance to chemotherapy [50]. Another study revealed that patients with AML had an overexpression of HO-1 at both the gene and protein levels [51]. Research by Lin and colleagues indicated also that *HO-1* suppressed the apoptosis of the HL-60 and U937 AML cell lines through activating the JNK/c-JUN signaling pathway. Moreover, the silencing of *HO-1* in the mouse xenograft model prolonged their survival [51]. On the other hand, it has been shown that cultured leukemic cell lines, such as HL-60, U937, and K562, are characterized by a low expression of the *HO-1* gene [52]. In our conditions, kaempferol, in contrast to its glycoside derivatives, increased the expression of the *HO-1* gene in HL-60 cells (Figure 1). Moreover, we observed that a co-treatment of the cells with all the tested polyphenols and etoposide increased the expression level of the *HO-1* gene (Figure 1). Treatment with kaempferol also increased the *HO-1* gene expression and the HO-1 protein level in the rat adrenal pheochromocytoma cell line (PC12) [53].

Here, we showed that kaempferol alone and in combination with etoposide significantly increases the expression of the *NQO1* gene. Studies carried out on chronic myeloid leukemia K562 cells have shown that *NQO1* knockdown promoted DNA synthesis and cell growth [20]. In addition, Xiao and colleagues tested the samples of peripheral blood or bone marrow specimens obtained from patients suffering from chronic myeloid leukemia and showed that the frequency of loss-of-function mutations of the *NQO1* gene was higher than that in healthy individuals. The increased expression of *NQO1* was also associated with the suppression of HL-60 cell proliferation [19]. Limonin, which is a secondary metabolite belonging to the tetracyclic triterpenoids, improved the activity of NQO1 in HL-60 cells and promoted apoptosis [54]. In our previous work, we demonstrated that a co-treatment of HL-60 cells with etoposide and kaempferol led to a decrease of cell viability measured by the resazurin reduction test [29]. Probably, the increase in cytotoxicity that we observed previously could be related to the increased expression of the *NQO1* gene in HL-60 cells. All three tested kaempferol derivatives did not influence the cytotoxic effect of etoposide on HL-60 cells [29]. This is in line with the results describing *NQO1* gene expression in cells co-treated with kaempferol derivatives and etoposide (Figure 1). P2 and P5 did not change the level of this gene in comparison to the cells incubated only with etoposide (Figure 1). We observed only a slight increase in *NQO1* gene expression in the case of the P7 derivative at its highest concentration. Interestingly, mangiferin, which is a glucosylxanthone extracted from plants of the *Anacardiaceae* and *Gentianaceae* families, activated the NFE2L2-ARE pathway in HL-60 cells and increased *NQO1* transcription, but a co-treatment of these cells with 50-µM mangiferin and 4–500-µg/mL etoposide did not change the etoposide-induced cytotoxicity and apoptosis [55].

We observed that the incubation of HL-60 cells with 1-µM etoposide leads to an increase in the level of total intracellular glutathione (Figure 3). The increase in the level of total glutathione in cells incubated with the drug may be caused by the cellular response to the action of the oxidative stress-inducing factor, which is etoposide. On the other hand, it was shown that etoposide can act as both an antioxidant and a pro-oxidant in HL-60 cells [56]. The phenolic moiety of etoposide acts as an effective free radical scavenger, accounting for its antioxidant action, whereas the one-electron oxidation of etoposide by free radical scavenging and/or by myeloperoxidase (MPO) results in a phenoxyl radical with low reactivity toward lipids, but its high reactivity toward thiols is a determinant of its pro-oxidant effects in HL-60 cells. Moreover, the treatment of HL-60 cells with etoposide increases the expression of the *HO-1* gene and enhances the activity of SOD enzymes, which are also responsible for protecting the cell against ROS (Figure 1 and Figure 2). When HL-60 cells were incubated with another drug used in the treatment of AML, arsenic trioxide, an increase in the glutathione content in the cells was also observed after 24 h [57]. Moreover, arsenic trioxide activates *NFE2L2* and its downstream target genes *HO-1* and *NQO1*. We showed that both kaempferol and its glycoside derivatives do not affect the level of total glutathione in HL-60 cells (Figure 3). Similar results to those obtained by us have been obtained in studies on Caco-2 cells. Kaempferol at the concentration of 10 µg/mL did not change the ratio of GSH/GSSG in this cell line [45]. Kaempferol increased the activity of glutathione peroxidase (GSH-Px) and glutathione reductase (GSH-Rx) in the 3T3-L1 cell line but did not increase the level of GSH measured in these cells after 24 h of incubation [46].

We noticed that kaempferol and derivative P7 increased the level of total glutathione in HL-60 cells treated with etoposide (Figure 3). It is probably caused by the stimulation of enzymes involved in the synthesis of glutathione, such as γ-glutamate-cysteine ligase (GCL) [33]. Moreover, we did not observe any changes in the cells co-incubated with P2 or P5 and etoposide. In our previous work, we observed that kaempferol and its glycoside derivative P5 did not affect the level of apoptosis induced by 24 h of incubation with etoposide in HL-60 cells [29]. Additionally, we noticed that derivatives P2 and P7 in the concentration of 50 µg/mL slightly decreased the apoptosis induced by etoposide. This is in line with the results obtained by incubating myeloid leukemia cell lines with topoisomerase II inhibitors (etoposide and doxorubicin) and polyphenols [6]. Emodin, rhein, and *cis*-stilbene, which did not affect the level of glutathione or lead to its increase, also reduced the level of apoptosis in cells treated with topoisomerase II inhibitors. On the other hand, quercetin and apigenin, in combination with etoposide or doxorubicin, caused a reduction of the glutathione level and the induction of apoptosis. In other studies, curcumin decreased the level of GSH in HL-60 cells treated with etoposide, and it also increased apoptosis [58].

The ability of kaempferol to increase the glutathione level in cells incubated with an anticancer drug has also been reported in normal cells. Research conducted on human umbilical vein endothelial cell HUVECs showed that kaempferol protected these cells from doxorubicin-induced endotheliotoxicity through decreasing ROS generation and maintaining the GSH/GSSG balance [59]. Studies on rats have shown that kaempferol increases the intracellular level of GSH in the hippocampi of both control and CdCl_2_ (cadmium chloride)-treated animals through the upregulation of NFE2L2 and activation of AMPK [60].

## 5. Conclusions

Kaempferol, due to its antioxidant properties, can directly influence the antioxidant status of cancer cells. Our studies have shown that this polyphenol may influence the oxidative balance indirectly by affecting the expression of antioxidant genes and proteins. We have shown that kaempferol and its glycoside derivatives did not change the expression of the *NFE2L2* gene.

Etoposide is an anticancer drug that can induce oxidative stress in cancer cells and lead to their death. We have shown that kaempferol and its glycoside derivatives isolated from the aerial parts of *Lens culinaris* Medik. increase the expression of antioxidant genes, especially the *HO-1* gene, SOD activity, and glutathione level in cells treated with etoposide. It seems that the phenolic acid residues attached to the same skeleton are responsible for the differences in the action of kaempferol derivatives, i.e., the caffeic acid residue in P2, the *p*-coumaric acid residue in P5, and the ferulic acid residue in P7. Our results indicated that kaempferol and its derivatives may modulate the activity of etoposide and affect the effectiveness of anticancer therapy based on this drug.

## Data Availability

Not applicable.

## Supplementary Materials

The following supporting information can be downloaded. Table S1: Gene expression changes in HL-60 cells treated with etoposide and kaempferol or its glycoside derivatives.
