# Supplementary material for: Effect of Kaempferol and Its Glycoside Derivatives on Antioxidant Status of HL-60 Cells Treated with Etoposide
Source: Molecules. 2022 Jan 6;27(2):333. doi: 10.3390/molecules27020333 (PMC8777684; doi:10.3390/molecules27020333)
Supplement: Supplementary file 1 [file molecules-27-00333-s001.zip › molecules-1500224-supplementary.pdf]

Supplementary Materials

Effect of Kaempferol and Its Glycoside Derivatives on Antioxidant Status of HL-60 Cells Treated with Etoposide

Magdalena Kluska[1], Michał Juszczak[1], Jerzy Żuchowski[2], Anna Stochmal[2] and Katarzyna Woźniak[1*]

1 Department of Molecular Genetics, Faculty of Biology and Environmental Protection, University of Lodz, 90-236 Lodz, Poland

2 Department of Biochemistry and Crop Quality, Institute of Soil Science and Plant Cultivation, State Research Institute, 24-100 Pulawy, Poland

* Correspondence: katarzyna.wozniak@biol.uni.lodz.pl; Tel.: +48-42-635-47-76; Fax: +48-42-635-44-84

Table S1. Gene expression changes in HL-60 cells treated with etoposide and kaempferol or its glycoside derivatives.

Treatment/Concentration	Relative gene expression (fold change)				
	NFE2L2	***HO-1***	***NQO1***	***SOD1***	***SOD2***
Ctrl	1 ± 0.16	1 ± 0.11	1 ± 0.16	1 ± 0.16	1 ± 0.27
K 10 µg/mL	0.77 ± 0.25	1.39 ± 0.18 **	1.6 ± 50.16 **	1.22 ± 0.1	1.2 ± 0.11
K 50 µg/mL	0.98 ± 0.15	2.52 ± 0.42 ***	2.3 ± 0.27 ***	1.42 ± 0.23 *	1.54 ± 0.25 *
P 2 10 µg/mL	0.8 5± 0.07	0.79 ± 0.17	1.18 ± 0.52	1.5 ± 0.28 *	1.08 ± 0.33
P 2 50 µg/mL	0.9 ± 0.27	1.09 ± 0.14	1.32 ± 0.13 *	1.18 ± 0.13	1.25 ± 0.09
P 5 10 µg/mL	0.91 ± 0.13	0.99 ± 0.12	1.24 ± 0.32	1.99 ± 0.24 ***	1.57 ± 0.35 *
P 5 50 µg/mL	0.96 ± 0.07	1.1 ± 0.29	1.3 ± 0.15 *	1.14 ± 0.15	1.1 ± 0.14
P 7 10 µg/mL	0.92 ± 0.14	1 ± 0.24	1.27 ± 0.3	1.3 ± 0.33	1.12 ± 0.23
P 7 50 µg/mL	1.08 ± 0.42	1.06 ± 0.25	1.75 ± 0.2 **	1.72 ± 0.14 ***	1.65 ± 0.23 *
E 1 µM	1.13 ± 0.27	4.87 ± 0.18 ***	1.14 ± 0.26	1.01 ± 0.1	1.01 ± 0.09
E 1 µM + K 10 µg/mL	0.98 ± 0.28	9.49 ± 0.51 *** ###	1.51 ± 0.51	1.68 ± 0.52 * #	1.72 ± 0.53 * #
E 1 µM + K 50 µg/mL	1.06 ± 0.15	9.33 ± 0.35 *** ###	1.84 ± 0.38 ** #	1.41 ± 0.36	1.72 ± 0.37 * ##
E 1 µM + P 2 10 µg/mL	1.08 ± 0.44	4.83 ± 0.28 ***	1.36 ± 0.37	1.21 ± 0.09 #	0.83 ± 0.55
E 1 µM + P 2 50 µg/mL	1.28 ± 0.17	6.62 ± 0.28 *** ###	1.21 ± 0.37	1.46 ± 0.09 ** ###	1.89 ± 0.55 * #
E 1 µM + P 5 10 µg/mL	1.07 ± 0.16	5.45 ± 0.1 *** ###	1.28 ± 0.24	1.11 ± 0.08	0.9 ± 0.32
E 1 µM + P 5 50 µg/mL	1.09 ± 0.11	9.97 ± 0.42 *** ###	1.69 ± 0.41 *	1.34 ± 0.35	1.59 ± 0.4 #
E 1 µM + P 7 10 µg/mL	1.22 ± 0.16	5.29 ± 0.17 *** #	1.3 ± 0.13 *	1.43 ± 0.18 * ##	1.18 ± 0.15
E 1 µM + P 7 50 µg/mL	1.19 ± 0.23	5.49 ± 0.22 *** ##	1.55 ± 0.12 ** #	1.28 ± 0.17 #	1.38 ± 0.13 * ##

NFE2L2 - nuclear factor erythroid 2-related factor 2, *HO-1* - heme oxygenase-1, *NQO1* - NAD(P) H:quinone oxidoreductase 1, *SOD1* - superoxide dismutase 1, *SOD2* - superoxide dismutase 2. Relative expression of genes in HL-60 cells incubated for 24 h at 37 °C with 10-50 µg/mL kaempferol (K), P2, P5, P7 and/or 1 µM etoposide (E). The table shows mean results ± SD, $n = 4$; * $p < 0.05$, ** $p < 0.01$, *** $p < 0.001$ vs. control (Ctrl); # $p < 0.05$, ## $p < 0.01$, ### $p < 0.001$ vs. etoposide (E).
